# Preventing Posterior Collapse with DVAE for Text Modeling

**DOI:** 10.3390/e27040423

**Published:** 2025-04-14

**Authors:** Tianbao Song, Zongyi Huang, Xin Liu, Jingbo Sun

**Affiliations:** 1 School of Computer and Artificial Intelligence, Beijing Technology and Business University, Beijing 100048, China; songtianbao@btbu.edu.cn (T.S.); maturehzy@st.btbu.edu.cn (Z.H.); 2 The 15th Research Institute of China Electronics Technology Group Corporation, Beijing 100083, China; threetwoli@163.com; 3 School of Information Engineering, Minzu University of China, Beijing 100081, China; 4 National Language Resource Monitoring and Research Center of Minority Language, Minzu University of China, Beijing 100081, China

**Keywords:** variational autoencoder, text modeling, posterior collapse, latent variable

## Abstract

This paper introduces a novel variational autoencoder model termed DVAE to prevent posterior collapse in text modeling. DVAE employs a dual-path architecture within its decoder: path A and path B. Path A makes the direct input of text instances into the decoder, whereas path B replaces a subset of word tokens in the text instances with a generic unknown token before their input into the decoder. A stopping strategy is implemented, wherein both paths are concurrently active during the early phases of training. As the model progresses towards convergence, path B is removed. To further refine the performance, a KL weight dropout method is employed, which randomly sets certain dimensions of the KL weight to zero during the annealing process. DVAE compels the latent variables to encode more information about the input texts through path B and fully utilize the expressiveness of the decoder, as well as avoiding the local optimum when path B is active through path A and the stopping strategy. Furthermore, the KL weight dropout method augments the number of active units within the latent variables. Experimental results show the excellent performance of DVAE in density estimation, representation learning, and text generation.

## 1. Introduction

Variational autoencoder (VAE) [1,2] is a widely used generative framework that combines deep latent variable models with amortized variational inference techniques. Recent years have witnessed great success for VAE as a generative model in various challenging domains, including natural language processing [3,4,5]. A key component of VAE is the latent variable with distributional properties. VAE can explicitly model high-level linguistic and semantic features of texts using this latent variable [6], and the resulting latent variables can be viewed as representations of the input texts. In addition, sampling from the prior distribution of the latent variables and performing simple deterministic decoding through the decoder in VAE can generate diverse grammatical texts.

However, the amount of information stored in the latent variables is strongly related to the expressiveness of the decoder in VAE. If the decoder is expressive enough to model the data, VAE tends to ignore the latent variables, resulting in trivial posteriors that are almost identical to the prior. This notorious problem is known as KL (Kullback–Leibler divergence) vanishing [6,7], KL collapse [8], latent variable collapse [9], or posterior collapse [10].

The posterior collapse problem is first detailed in [6], where the VAE employs an LSTM (long-short-term memory) decoder for text modeling. They proposed two methods to address this issue. The first method is referred to as KL cost annealing in [6]. In this method, the KL regularization term in the objective is assigned a KL weight that increases gradually from 0 to 1. The second method is named word dropout and historyless decoding. In this method, some or all of the conditioned-on word tokens in the decoder are removed. Although some subsequent works have found that the KL cost annealing method is not suitable for complex text datasets with large LSTM decoders [11] and the optimal annealing scheme varies with different datasets and model architectures [12], it is still widely used and serves as the basis for many other methods. However, the word dropout and historyless decoding method has not received much attention and has not been well studied. A major concern may be that the word dropout and historyless decoding method does not make full use of the expressive decoder. A VAE without posterior collapse is not necessarily a good model, because it should also be able to perform accurate data density estimations, learn meaningful representations, and generate high-quality results. However, failing to fully exploit the decoder will discount the performance of VAE in these aspects.

This paper presents a novel model to address the limitations of the word dropout and historyless decoding method. In this model, each text instance will be fed into the decoder twice, denoted by path A and path B, respectively. In path A, the original word tokens are fed to the decoder, but in path B, the word tokens are randomly replaced with the generic unknown word token <u> in a certain proportion before being fed to the decoder. The decoders of path A and path B share parameters, as well as latent variables obtained by the encoder. The mean of the cross-entropy obtained by path A and path B is used as the reconstruction term in the objective. When the model approaches convergence, remove path B and revert it back to the basic VAE. In addition, during the KL cost annealing process, the KL weights for a certain proportion of the dimensions in the latent variable are set to zero, in order to promote the number of active units in the latent variable. The model is named DVAE (dropout variational autoencoder). It is important to point out that the dropout in DVAE is applied to word tokens and KL weights rather than features, and there are not two decoders, but only two paths through the decoder after processing the input tokens differently. The contributions of this paper can be summarized as follows.

- We take the word dropout and historyless decoding method and the KL cost annealing method a step further, proposing the DVAE model to solve the posterior collapse problem in VAE for text modeling without compromising the expressiveness mining and utilization of the decoder.
- We experiment with DVAE in various settings and report improved results with respect to multiple evaluation metrics.

## 2. Preliminary

### 2.1. The VAE Model

Consider a latent variable model defined by a joint distribution $p_{\theta }\left(x,z\right)=p_{\theta }\left(x|z\right)p\left(z\right)$ between the data space x∈X and the latent space z∈Z. Given the data *x* under the empirical distribution $p_{X}\left(x\right)$, we would like to train the model by maximizing the marginal likelihood:

(1) $$E_{p_{X}\left(x\right)}\left[\log p_{\theta }\left(x\right)\right]=E_{p_{X}\left(x\right)}\left[\log\int_{Z}p_{\theta }\left(x|z\right)p\left(z\right)dz\right].$$

To calculate this intractable marginal likelihood, VAE [1,2] uses amortized variational inference and introduces the distribution $q_{\phi }\left(z|x\right)$ parameterized by a neural network $f_{\phi }\left(\cdot \right)$ to approximate the true posterior. Then, instead, VAE turns to optimize a tractable evidence lower bound (ELBO):

(2) $$L_{ELBO}=E_{p_{X}\left(x\right)}\left[\;\underset{reconstruction\;term}{\underbrace{E_{q_{\phi }\left(z|x\right)}\left[\log p_{\theta }\left(x|z\right)\right]}}-\underset{KL\;regularization\;term}{\underbrace{D_{KL}\left[q_{\phi }\left(z|x\right)∥p\left(z\right)\right]}}\;\right],$$

where the prior p(z) is typically assumed as the multivariate Gaussian distribution N(0,I), $q_{\phi }\left(z|x\right)$ is parameterized as a multivariate Gaussian distribution $N(\mu ,\;\sigma^{2})$ by the neural network $f_{\phi }\left(\cdot \right)$, and $p_{\theta }\left(x|z\right)$ is parameterized by another neural network $f_{\theta }\left(\cdot \right)$.

VAE can also be considered as a regularized version of the standard autoencoder (AE) [6]. It has the same encoder–decoder structure as AE but replaces deterministic latent variables with distribution-based ones. The encoder in VAE maps the input data to a probabilistic latent variable *z* under the distribution $q_{\phi }\left(z|x\right)$ that is restricted by p(z), and the decoder reconstructs the input data using *z* according to $p_{\theta }\left(x|z\right)$. The first part of Equation (2) is the reconstruction term that forces VAE to reconstruct the input data, and the second part is the KL regularization term that forces the posterior distribution $q_{\phi }\left(z|x\right)$ to match the prior p(z).

Due to the sequential nature of text, VAE typically employs an autoregressive decoder to model text data. For a text sequence $x=[x_{1},x_{2},x_{3},\ldots ,x_{T}]$, the decoder in VAE predicts each token $x_{t}$ conditioned on previously generated tokens and the latent variable *z*:

(3) $$p_{\theta }\left(x|z\right)=\prod_{t=1}^{T}p_{\theta }\left(x_{t}|x_{<t},z\right).$$

### 2.2. Posterior Collapse of VAE

VAE learns distribution-based latent variables as representations for input data and constrains these distributions by the KL regularization term. This constraint, on the one hand, mines the similarities between the input data to construct a continuous latent space; on the other hand, it ensures that the latent variables sampled from the prior distribution can be decoded to generate new reasonable data. From this point of view, the smaller KL regularization term is better, that is, the more the posterior distributions approach the prior, the better. However, when the posterior distributions are too close to the prior, for each data instance *x*, there exists $q_{\phi }\left(z|x\right)\approx p\left(z\right)$, that is, the KL regularization term in Equation (2) vanishes to 0; this is the posterior collapse problem. When posterior collapse occurs, the posterior distributions do not depend on the input data, and the latent variables hardly capture any faithful information about the data.

The posterior collapse problem is more common when VAE employs autoregressive models as the decoder and is especially evident when modeling discrete data such as text. When posterior collapse occurs in text modeling, Equation (3) turns to the following form:

(4) $$p_{\theta }\left(x|z\right)=\prod_{t=1}^{T}p_{\theta }\left(x_{t}|x_{<t}\right),$$

which means VAE degenerates to autoregressive and is ready to attain higher optimization objective benefits without depending on latent variable *z*. In Equation (2), the reconstruction term is basically independent of the latent variable *z*, and under the constraint of the KL regularization term, the posterior distribution $q_{\phi }\left(z|x\right)$ will approach the prior p(z) infinitely to make the optimization objective optimal. However, in this case, VAE actually only achieves a local optimum, and the latent variable fails to support VAE for effective density estimation; VAE fails to learn effective representations for the texts. New data generated from the prior distribution will severely lack diversity.

Bowman et al. [6] proposed the word dropout and historyless decoding method to mitigate the posterior collapse of VAE in text modeling. This method randomly removes some or all of the conditioned-on word tokens in the decoder. Then, Equation (3) turns to:

(5) $$p_{\theta }\left(x|z\right)=\prod_{t=1}^{T}p_{\theta }\left(x_{t}|\tilde{x_{<t}},z\right),$$

where $\tilde{x_{<t}}$ means a proportion of the word tokens $x_{<t}$ are replaced with the generic unknown word token <u>. Due to the absence of some word tokens in $\tilde{x_{<t}}$, the VAE decoder cannot obtain enough information from $\tilde{x_{<t}}$ to predict $x_{t}$ and is forced to use the information in the latent variable *z*. Therefore, even though the KL regularization term in Equation (2) still pushes the posterior distribution $q_{\phi }\left(z|x\right)$ to the prior p(z), VAE must balance this with the reconstruction term to optimize the overall objective, thus mitigating the posterior collapse problem.

However, the word dropout and historyless decoding method alleviate the posterior collapse problem at the cost of the decoder performance, as it fails to adequately capture the local dependencies among word tokens. Probably for this reason, this method has not been widely used or further studied.

## 3. Related Work

Many methods have been proposed to mitigate the posterior collapse problem of VAE. The most popular among these is probably the KL cost annealing method [6], in which the weight of the KL regularization term is gradually increased according to a monotonic annealing schedule. In this class of methods, two other related schedules are the constant schedule and the cyclical annealing schedule [8]. The constant schedule mainly refers to the β regularized version of VAE, which re-weights the KL regularization term using a constant β [13,14,15,16]. By setting β>1, VAE can learn disentangled latent variables, which is the primary goal in [13,15,16]. Setting β<1 can solve the posterior collapse problem, but setting β≠1 results in an improper statistical model [14]. The cyclical annealing schedule [8] repeats the process of increasing the weight of the KL regularization term multiple times, which can leverage the latent variables of previous cycles as warm restarts to learn more meaningful variables. This class of methods can also be seen as a trade-off between reconstruction and compression. In this view, eVAE [7] incorporates and integrates variational evolutionary learning and variational information bottleneck into VAE to achieve better optimum exploration and the trade-off between representation compression and generation fitting.

The second class of methods focuses on the KL regularization term to prevent it from being too small. The free-bits method [17] replaces the KL regularization term with a hinge loss term and stops optimization when the value of the term is lower than a threshold. Then, to solve the gradient discontinuities of free-bits, Pelsmaeker and Aziz [18] proposed the minimum desired rate technique to attain ELBO at a pre-specified rate. BN-VAE [19] keeps the expectation of the KL regularization term positive by batch normalization on the parameters of the approximate posteriors for latent variables. Shen et al. [20] extended BN-VAE with dropout on the variances of posteriors to learn a more diverse and less uncertain latent space. CEAE [10] replaces BN-VAE with a similar deterministic variant to improve performance in language modeling. δ-VAE [21] constrains the mean and variance of the posterior to have a minimum distance to the prior. Some methods use other distributions as the prior instead of Gaussian, such as the von Mises–Fisher distribution [22,23] and the uniform distribution [24,25], by which the KL regularization term is independent of the data instances.

The third class of methods is to enforce the relation between latent variables and input data through mutual information (MI)-based terms in the objective. InfoVAE [26] adds a KL divergence term between aggregated posteriors and priors on latent variables to the objective and controls MI together with the original KL regularization term. The Fisher autoencoder [27] implicitly controls the MI between latent variables and input data by setting the appropriate Fisher information constraint. MAE [28] adds a mutual posterior-divergence regularization to the objective, which has a similar goal to MI.

Another class of methods is to strengthen the encoder or weaken the decoder. To strengthen the encoder, SA-VAE [11] uses amortized variational inference to initialize VAE and then performs stochastic variational inference to refine them. He et al. [12] proposed to aggressively optimize the encoder before performing each update to the model. EM-VAE [29] links this aggressive training scheme with the expectation–maximization (EM) framework and uses an EM-type training algorithm that ensures a controllable optimization process. Li et al. [30] proposed to initialize the encoder with a pre-trained one from an autoencoder and continue training using the free-bits method. Weakening the decoder is closely related to our method, and the most relevant is the word dropout and historyless decoding method [6] that has been introduced in previous sections. Some other methods replace the autoregressive network in the decoder with a convolutional neural network [31,32]. The method in [33] introduced another regularization term based on fraternal dropout in the objective that forces the hidden states computed by the decoder to be similar with different masked input words but similar latent variables. The mask here is applied at the word embedding layer, that is, applying dropout to the word embedding. Unlike it, our method applies the mask at the word token level, replacing some tokens with the generic <u>. Compared to these methods, our method is able to exploit the expressiveness of the autoregressive decoder more fully.

## 4. Model

In view of the limitations of the word dropout and historyless decoding method, this paper presents a novel model, DVAE, in the hope of preventing posterior collapse without compromising the extraction and utilization of the expressive decoder. In Section 4.1, we delineate the model architecture and the objective of DVAE. Then, we introduce the path-stopping strategy of DVAE in Section 4.2. In Section 4.3, we detail the KL weight dropout method based on KL cost annealing.

### 4.1. Model Architecture

**Figure 1:**
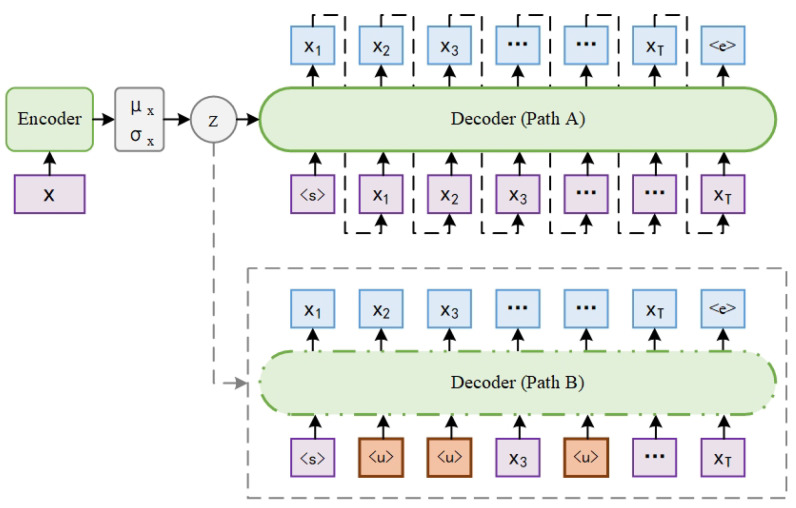
The overall architecture of DVAE. The decoders of path A and path B share parameters, as well as latent variables obtained by the encoder.

Figure 1 shows the model architecture of DVAE. It consists of an encoder and a decoder, the same as the basic VAE. Given a text instance $x=[x_{1},x_{2},x_{3},\ldots ,x_{T}]$, the encoder $Enc_{\phi }\left(\cdot \right)$ parameterizes the posterior distribution $q_{\phi }\left(z|x\right)$ as an *n*-dimensional Gaussian distribution $N(\mu_{x},\;\sigma_{x}^{2})$ with mean $\mu_{x}$ and diagonal covariance $\sigma_{x}^{2}$. Using $\mu_{x}$ and $\sigma_{x}$, a latent variable *z* is obtained from $z=\mu_{x}+\sigma_{x}\cdot \varepsilon$, a differentiable transformation of the noise variable ε sampled from the Gaussian distribution N(0,I), according to the reparameterization trick [1,2].

The decoder $Dec_{\theta }\left(\cdot \right)$ reconstructs the text instance *x* based on *x* and the latent variable *z*. Unlike the basic VAE, there are two paths in the decoder, which are denoted by path A and path B for description convenience. In path A, the sequence of word tokens $x=[x_{1},x_{2},x_{3},\ldots ,x_{T}]$ is fed into the decoder. In path B, a percentage α of the word tokens within the sequence are randomly selected and replaced with the generic unknown word token <u> before being fed into the decoder. For example, the sequence of word tokens after replacement may be $\tilde{x_{\alpha }}=\left[<u>,<u>,x_{3},<u>,\ldots ,x_{T}\right]$, as shown in Figure 1.

Path B is not always active in the decoder, which will be discussed in detail in Section 4.2. When both path A and path B are active, the objective to optimize in DVAE is as follows:

(6) $$\begin{array}{cc} \; & L_{D}\left(x,\alpha ;\;\phi ,\;\theta \right)=\\ & E_{p_{X}\left(x\right)}\left[\;\underset{reconstruction\;term}{\underbrace{\frac{1}{2}E_{q_{\phi }\left(z|x\right)}\left[\underset{recon\;of\;path\;A}{\underbrace{\log\prod_{t=1}^{T}p_{\theta }\left(x_{t}|x_{<t},z\right)}}+\underset{recon\;of\;path\;B}{\underbrace{\log\prod_{t=1}^{T}p_{\theta }\left(x_{t}|\tilde{x_{<t,\alpha }},z\right)}}\right]}}-\underset{KL\;regularization\;term}{\underbrace{D_{KL}\left[q_{\phi }\left(z|x\right)∥p\left(z\right)\right]}}\;\right], \end{array}$$

where $\tilde{x_{<t,\alpha }}$ denotes that α percent of the word tokens in $x_{<t}$ are replaced with <u>. When only path A is active, the objective of DVAE is the same as that of basic VAE for text modeling, as follows:

(7) $$L_{D}\left(x;\;\phi ,\;\theta \right)=E_{p_{X}\left(x\right)}\left[\;\underset{reconstruction\;term}{\underbrace{E_{q_{\phi }\left(z|x\right)}\left[\underset{recon\;of\;path\;A}{\underbrace{\log\prod_{t=1}^{T}p_{\theta }\left(x_{t}|x_{<t},z\right)}}\right]}}-\underset{KL\;regularization\;term}{\underbrace{D_{KL}\left[q_{\phi }\left(z|x\right)∥p\left(z\right)\right]}}\;\right].$$

Posterior collapse is a multifaceted issue that can be examined from various angles. From the perspective of model collapse and inference collapse, posterior collapse often arises when the optimization of the encoder lags that of the decoder [12]. This lagging problem initially leads to inference collapse, which subsequently triggers model collapse and ultimately culminates in posterior collapse. Addressing this issue from this perspective typically involves either weakening the decoder or strengthening the encoder.

In DVAE, path B weakens the decoder by randomly dropping word tokens from the input text. The sequence of word tokens $\tilde{x_{<t,\alpha }}$ containing α percent of <u> cannot provide enough information for the decoder to use. In order to obtain a desirable value for the recon of the path B term in Equation (6), the model has to encode essential information about the text instance *x* in the latent variable *z*.

While this mechanism can address the issue of posterior collapse, it does not resolve the problem of the weakened decoder. To address this limitation, path A is also introduced. In path A, the decoder has access to the entire input text and can fully exploit its autoregressive nature to capture local dependencies between word tokens. Since path A and path B merely represent different pathways of the same decoder, their decoders share identical parameters. As a result, path B can still benefit from the local information captured by path A, even though it cannot directly see part of the input text. This design not only mitigates the performance loss of the decoder but also ensures that the learned latent variables are compatible with the state of the decoder that has access to the full input text. As a result, the model is less likely to fall into the local optimum, achieving a better balance between preventing posterior collapse and maintaining decoder performance.

### 4.2. Stopping Strategy

While training according to Equation (6) with both paths A and B can mitigate the loss of expressiveness in the decoder, continuous training under this framework will still have an adverse effect. Therefore, in this section, we discuss the stopping strategy for path B.

We tried three stopping strategies. The first strategy is to train the model solely with path B until mutual information $I_{q}\left(x,z\right)$ between *x* and *z* under $q_{\phi }\left(x,z\right)$ [34] stops climbing, after which only path A is activated and the model is trained according to Equation (7). This strategy solves the posterior collapse problem but does not achieve the desired result in terms of density estimation. When the percentage α of the word token <u> is relatively large, the model gets a good reconstruction term, but the KL regularization term is very large, so the overall objective cannot reach the ideal level. We suspect that this is because the decoder has been trained without most of the word tokens for a long time, which leads the model to a local optimum that relies more on latent variables and less on local word dependencies. When α is relatively small, although the model solves the posterior collapse problem, the information encoded in the latent variables is not enough, and the overall objective is also not ideal.

The second strategy is to train the model according to Equation (6) with both path A and path B until $I_{q}\left(x,z\right)$ stops climbing, then just set path A and train the model according to Equation (7). However, in the presence of both path A and path B, the mutual information $I_{q}\left(x,z\right)$ always fluctuates up and down, and it is difficult to find a moment when $I_{q}\left(x,z\right)$ stops climbing.

The third strategy is similar to the second one, except that instead of using mutual information as the criterion for stopping path B, it stops path B when the model approaches convergence. In the experiments of Section 5, we chose to stop path B after the last decay of the learning rate. In this stopping strategy, when both path A and path B exist, path B forces the model to encode more information in the latent variables, and for path A, in addition to enabling the decoder to mine local word dependency information, it also enables the latent variables to be strengthened by path B to maintain coordination with the expressive decoder. When the model approaches convergence and stops path B, it is fine-tuned under the existing training state according to the objective of the basic VAE. Using this stopping strategy, the training procedure for DVAE is shown in Algorithm 1.
**Algorithm 1** Training Procedure for DVAE1:Initialize ϕ, θ, and α2:pathB←True3:**while** not convergence **do**4:    **for** *x* in mini-batches **do**5:        $\mu_{x},\;\sigma_{x}^{2}=Enc_{\phi }\left(x\right)$6:        Sample $z∼N(\mu_{x},\;\sigma_{x}^{2})$7:        **if** pathB **then**8:           Reconstruct *x* from $Dec_{\theta }\left(z,x\right)$9:           Reconstruct *x* from $Dec_{\theta }\left(z,\tilde{x_{\alpha }}\right)$10:         $g_{\phi ,\;\theta }\leftarrow {▽}_{\phi ,\;\theta }L_{D}\left(x,\alpha ;\;\phi ,\;\theta \right)$11:      **else**12:         Reconstruct *x* from $Dec_{\theta }\left(z,x\right)$13:         $g_{\phi ,\;\theta }\leftarrow {▽}_{\phi ,\;\theta }L_{D}\left(x;\;\phi ,\;\theta \right)$14:      **end if**15:      Update ϕ,θ according to $g_{\phi ,\;\theta }$16:  **end for**17:  Update pathB according to the stopping strategy18:**end while**

### 4.3. KL Weight Dropout

When no posterior collapse occurs in VAE, the most intuitive indication is that the KL regularization term does not diminish to an insignificantly small value. However, a KL regularization term that is not too small is not a sufficient condition for a good VAE.

Consider the mean $\mu =[\mu_{1},\mu_{2},\ldots ,\mu_{i},\ldots ,\mu_{D}]$ of the posterior distributions for latent variables. First, assuming that the KL regularization term follows a distribution across the entire dataset, then $E\left[KL\right]\geq \sum_{i=1}^{D}\left(Var\left[\mu_{i}\right]+E^{2}\left[\mu_{i}\right]\right)$ [19,20]. If the expectation $E[\mu_{i}]$ for each dimension or even part of the dimensions can be kept positive, the KL regularization term can get a positive lower bound. However, if the variance $Var[\mu_{i}]$ for each dimension approaches zero, this positive KL regularization term cannot make VAE escape from the posterior collapse. So, it is important that the μ for different data instances have a proper degree of discrimination in the latent space, rather than a higher KL. Second, some methods force the KL regularization term to be greater than a certain positive constant [19,20,21], which can make different data instances have discrimination in the latent space, but this coercive method may cause the model not to be able to obtain enough corresponding mutual information between the latent variables and the data. Third, when there is no posterior collapse, for the same KL value, the VAE training is more prone to gain by keeping $Var[\mu_{i}]$ not too close to zero in a few dimensions of μ, while the other dimensions all tend to zero; that is, only a few dimensions of the latent variables are active. However, the limited active units in the latent variables will affect the performance of VAE in density estimation, representation learning, and generation [35,36]. For the first two problems, DVAE sets path B with the goal of improving the information about the data in the latent variables to naturally increase the discrimination of different data instances in the latent space. For the third problem, we propose the KL weight dropout method.

In the traditional KL cost annealing method [6], the KL weight is a scalar value that serves as the coefficient of the KL regularization term in the objective. When the KL weight is relatively small, the model will not over-optimize the KL regularization term. This ensures that the KL value does not excessively approach zero, thereby preventing posterior collapse. However, compared to maintaining a certain distance between every dimension of the latent variables and the prior distribution N(0,1) to prevent the KL regularization term from becoming too close to zero, it is easier for model optimization to push only a few dimensions of the latent variables away from the prior N(0,1), while allowing the other dimensions to approach the prior [35,36]. As a result, the number of active units in the latent variables remains limited under this KL cost annealing method.

In the KL weight dropout method, the KL weight is a vector with the same dimensions as the latent variables, and each dimension follows the monotonic annealing schedule of the KL cost annealing method. Since both the posterior and prior distributions of the latent variables are isotropic Gaussian distributions, the KL divergence for each dimension can be calculated separately. This multidimensional KL weight is then used as the coefficient. In each training epoch, different dimensions of the KL weight are randomly set to zero, with the proportion controlled by the hyper-parameter λ. By doing so, different dimensions of the latent variable are exempt from the KL regularization term in different training epochs, thus enabling the model to have more active units in the latent variables that can encode information about the input data.

## 5. Experiment

### 5.1. Experimental Setup

We compared DVAE with the following models targeting the posterior collapse problem on text modeling benchmarks.

- LSTM language model.
  - **LSTM-LM**: the LSTM language model.
- Models using different annealing schedules.
  - **VAE**: the standard VAE with annealing [6].
  - **Cyclical**: VAE using the default cyclical annealing schedule in [8].
  - **β-VAE**: uses parameter β to reweight the KL regularization term [13].
- Models that focus on the KL regularization term to prevent it from being too small.
  - **FB**: VAE that stops optimization of the KL regularization term when it is lower than a threshold [17].
  - **BN-VAE**: controls the lower bound on the expectation of the KL regularization term using batch normalization [19].
  - **DU-VAE**: extends BN-VAE with dropout on the variances of posteriors to learn a more diverse and less uncertain latent space [20].
  - **δ-VAE**: constrains the minimum of KL regularization term by setting the mean and variance of posteriors in a specific range [21].
- MI-based models.
  - **MAE**: uses two parameters to control the diversity and smoothness of the latent space [28].
- Models that strengthen the encoder or weaken the decode.
  - **SA-VAE**: combines amortized variational inference and stochastic variational inference [11].
  - **Agg-VAE**: optimizes the encoder aggressively before performing each model update [12].
  - **EM-VAE**: links the aggressive training scheme of the encoder with the EM framework [29].
  - **CNN-VAE**: uses a convolutional neural network as the decoder [32].
  - **VAE-wh**: VAE using word dropout and historyless decoding [6].

The implementations follow the strong baselines: BN-VAE [19], DU-VAE [20], and Agg-VAE [12]. Both the encoder and the decoder use a one-layer LSTM with hidden size 1024. The dimension of the word embedding layer is 512. The LSTM layers and embedding layers are initialized with uniform distributions on [−0.01, 0.01] and [0.1, 0.1], respectively. Dropout with probability 0.5 is applied to both the word embeddings and the output features of the decoder. The dimension of the latent variable is 32, and after an affine transformation, it is used as the initial hidden state of the decoder. The latent variable is also concatenated with the input of the decoder. For all experiments, we used a Gaussian prior N(0,I) and applied a linear annealing strategy to increase the weight of the KL regularization term from 0 to 1 in the first 10 epochs. We utilized the SGD optimizer with 32 data instances per mini-batch and started with a learning rate of 1.0. We decayed the learning rate by 0.5 if the validation loss has not improved in 5 epochs and stopped training after 5 learning rate decays.

### 5.2. Density Estimation

For density estimation, we conducted experiments on two benchmark datasets: Yahoo and Yelp corpora [32]. Table 1 shows the results. NLL is the negative log-likelihood estimated by 500 importance-weighted samples [36] that provides a tighter lower bound compared to ELBO and shares the same information with perplexity. KL is the value of the KL regularization term $D_{KL}\left[q_{\phi }\left(z|x\right)∥p\left(z\right)\right]$. MI is the mutual information $I_{q}\left(x,z\right)$ between *x* and *z* under $q_{\phi }\left(x,z\right)$ and calculated by $I_{q}\left(x,z\right)=E_{p_{X}\left(x\right)}\left[D_{KL}\left[q_{\phi }\left(z|x\right)∥p\left(z\right)\right]\right]-D_{KL}\left[q_{\phi }\left(z\right)∥p\left(z\right)\right]$ [34]. AU is the number of active units [36] in the latent variables. The activity of a latent dimension *d* is measured as $A_{z_{d}}=Cov_{x}\left(E_{z_{d}∼q\left(z_{d}|x\right)}\left[z_{d}\right]\right)$. If $A_{z_{d}}>0.01$, the dimension *d* is considered active.

For the hyper-parameters α and λ in DVAE, we performed the experiments with a step size of 0.1 in the range of 0.1 to 0.9 and then reported the best results. KL weight dropout is only applied in the first 10 training epochs, that is, only when KL cost annealing is performed. For the word dropout rate in VAE-wh, we performed the experiments with a step size of 0.1 in the range of 0.1 to 0.5 and then reported the best results, as well as an additional result with a more ideal KL. For cyclical, we used the default cyclical annealing schedule in the first 10 epochs and did not search for the optimal annealing scheme. For other models, we reproduced the experiments with reference to the best parameters in the corresponding papers. We use NLL as the main metric in this part because it is the most direct metric to evaluate the performance of density estimation. Moreover, both the reconstruction and the KL values are included in NLL, and this combined value is important for VAE, which needs to make a trade-off between the reconstruction term and the KL regularization term.

DVAE achieves the best NLL on Yahoo and the same best NLL as BN-VAE and SA-VAE on Yelp. DVAE without KL weight dropout achieves the second and third-best NLL on Yahoo and Yelp, respectively, but the AU is not as good as that of DVAE. DVAE trained continuously with both path A and path B, without employing a stopping strategy, fails to achieve an ideal NLL. This indicates that employing dual paths coupled with the stopping strategy is effective in preventing posterior collapse and enhancing density estimation, and the KL weight dropout can further boost the active units in the latent variables, which in turn improves the NLL. VAE (w) fails to address the posterior collapse problem, suggesting that KL weight dropout alone is insufficient to resolve this challenge. VAE-wh with a word dropout rate of 0.1 achieves an acceptable NLL, but the KL value is poor, and posterior collapse occurs on Yelp. When the word drop rate is 0.3, although the KL is improved, the NLL is not good, indicating that the word dropout and historyless decoding method affects the density estimation ability of the model due to the weakening of the decoder. The competitive BN-VAE and DU-VAE have better AU compared to DVAE, but the batch normalization method applied to the mean of the posterior distribution in both models is equivalent to controlling the AU directly. This mandatory method creates a large $D_{KL}\left[q_{\phi }\left(z\right)∥p\left(z\right)\right]=KL-MI$, that is, a large KL between the aggregate posterior and the prior. Agg-VAE also achieved quite promising results but failed to yield good results in the experiments on another dataset, as described in Section 5.3.

### 5.3. Representation Learning

For representation learning, we trained a one-layer linear classifier using the means of posterior distributions on a downsampled version of the Yelp sentiment dataset [37]. Table 2 shows the density estimation performance of various models alongside their respective classification accuracies under varying quantities of labeled data. DVAE outperforms other models in terms of classification accuracy across all levels of labeled data, with the advantage becoming more pronounced as the amount of labeled data decreases.

BN-VAE and DU-VAE achieve competitive results. However, both models apply batch normalization to the mean of the posterior distribution, which is performed over mini-batches of texts, leading to fluctuating normalization scales. We hypothesize that this discrepancy affects the distribution of data in the latent space. To test this hypothesis, we conducted an intuitive experiment on a Mix dataset obtained by mixing the downsampled version of the Yelp sentiment dataset with a synthetic dataset. Following He et al. [12], the synthetic data are generated from a one-layer LSTM conditioned on 2-dimensional latent variables sampled from a Gaussian mixture distribution. This distribution consists of four mixture components with mean values at (−2.0, −2.0), (−2.0, 2.0), (2.0, −2.0), and (2.0, 2.0), each with unit variance. The Mix dataset comprises 37,500 data instances, which are divided into training/validation/test by 8/1/1. Within each part, the ratio of the instances from the downsampled version of the Yelp sentiment dataset and the synthetic dataset is 1:1.

**Figure 2:**
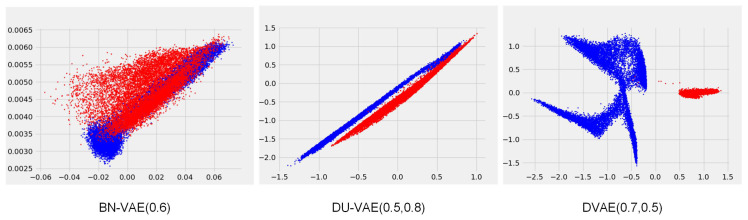
Visualization of the posterior means for various data instances learned by BN-VAE, DU-VAE, and DVAE. Blue points represent instances from the synthetic dataset and red points represent instances from the downsampled version of the Yelp sentiment dataset. The *x*-axis and *y*-axis represent the values of the first and second dimensions of the posterior means, respectively.

We trained DVAE, BN-VAE, and DU-VAE using this Mix dataset. The hidden size is 64. The dimensions of *z* and the input embedding layers are 2 and 32, respectively. The other settings are the same as those in Section 5.1. We partitioned the data instances into mini-batches in an extreme way, ensuring that the instances in the same mini-batch come from the same source, either Yelp or the synthetic dataset. Figure 2 visualizes the means of the approximate posteriors for various data instances learned by the three models. BN-VAE cannot distinguish the data instances from Yelp and the synthetic. DU-VAE is able to distinguish the data instances from Yelp and the synthetic due to the variance dropout in addition to batch normalization. DVAE excels further by not only distinguishing between Yelp and the synthetic but also by identifying instances generated from the four distinct Gaussian components within the synthetic dataset. Despite the atypical mini-batch partitioning during training, it is evident that both BN-VAE and DU-VAE have an impact on the distribution of data within the latent space.

### 5.4. Text Generation

This section discusses the evaluation results of the model generation performance. We generated two sets of texts using each model trained on the downsampled version of the Yelp sentiment dataset in Section 5.3.

The first set of texts is obtained by the following method, which we refer to as SampleGen, and it measures the ability of the models to generate texts by sampling the latent space.

1. Sample 5000 latent variables *z* from the prior distribution N(0,I) and then use them for greedy decoding.
2. Repeat the above step with 10 different random seeds, resulting in 50,000 generated texts.

The second set of texts is obtained by the following method, which we refer to as InterpolateGen, and it measures the smoothness of the latent space learned by the models.

1. Sample two latent variables z1 and z2 from the prior distribution N(0,I).
2. Obtain 5 latent variables by performing a linear interpolation between z1 and z2.
3. Repeat the above two steps 1000 times, resulting in 5000 latent variables.
4. Use these latent variables for greedy decoding, resulting in 5000 texts.
5. Repeat the above step with 10 different random seeds, resulting in 50,000 generated texts.

The evaluation results are shown in Table 3. GPT2-P is the perplexity of the generated texts evaluated using GPT-2. DIST represents the mean value of the proportions of distinct 1-grams, 2-grams, and 3-grams in the generated texts. MAUVE [38] measures the distribution closeness between the generated texts and human-written texts. A higher MAUVE score means that the model generates more human-like texts. We computed it using GPT-2. GPT2-P and DIST basically show a positive correlation, that is, better diversity leads to worse quality. Since MAUVE includes the evaluation of both diversity and quality, we divided the models into two groups based on this metric, as shown by the dashed line in Table 3, and marked the optimal results for each group. The upper group is not ideal on the MAUVE due to poor diversity, even if the quality is good. The lower group achieves close MAUVE in SampleGen and InterpolateGen, with the best result in DVAE. At the same time, DVAE also breaks the state of positive correlation between GPT2-P and DIST; that is, compared to BN-VAE and DU-VAE, DVAE has better GPT2-P when it achieves better or comparable DIST.

**Figure 3:**
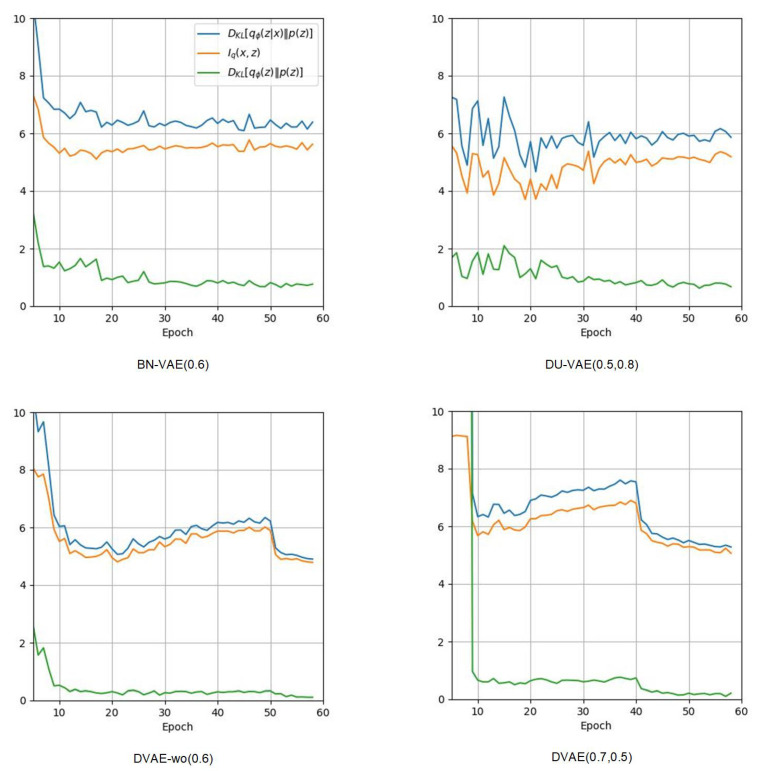
Visualization of the variation curves of $D_{KL}\left[q_{\phi }\left(z|x\right)∥p\left(z\right)\right]$, $D_{KL}\left[q_{\phi }\left(z\right)∥p\left(z\right)\right]$, and $I_{q}\left(x,z\right)$ throughout the training process.

Figure 3 illustrates the variation curves of $D_{KL}\left[q_{\phi }\left(z|x\right)∥p\left(z\right)\right]$, $D_{KL}\left[q_{\phi }\left(z\right)∥p\left(z\right)\right]$ and $I_{q}\left(x,z\right)$ throughout the training process. According to the equation of $I_{q}\left(x,z\right)$:

(8) $$I_{q}\left(x,z\right)=E_{p_{D}\left(X\right)}\left[D_{KL}\left[q_{\phi }\left(z|x\right)∥p\left(z\right)\right]\right]-D_{KL}\left[q_{\phi }\left(z\right)∥p\left(z\right)\right],$$

the MI $I_{q}\left(x,z\right)$ is the difference between two terms, where the first term is the KL regularization term, and the second term is the KL distance between the aggregated posterior $q_{\phi }\left(z\right)=E_{p_{D}\left(X\right)}\left[q_{\phi }\left(z|x\right)\right]$ and the prior p(z) (referred to as sampling distance henceforth). When generating new data samples, the first step is to sample a latent variable *z* from p(z), and the next step is to feed *z* into the decoder for generation. In order to have better generation results, a smaller sampling distance is better. Figure 3 shows that BN-VAE, DU-VAE, DVAE-wo, and DVAE are all able to prevent posterior collapse, but DVAE-wo and DVAE have lower sampling distances.

## 6. Conclusions

This paper focuses on the posterior collapse problem in VAE on text modeling when employing an autoregressive decoder. Starting from the limitations of the word dropout and historyless decoding method, we propose DVAE. DVAE employs a dual-path architecture within its decoder to compel the latent variables to encode more information about the input data. The design of a path-stopping strategy enables the model to fully leverage the expressiveness of the decoder and prevents the model from converging to a local optimum. Additionally, a KL weight dropout method is utilized to increase the number of active units within the latent variables. Experimental results show the excellent performance of DVAE in density estimation, representation learning, and text generation. Compared to the most competitive models, DVAE does not affect the distribution of data instances in the latent space and possesses a smaller KL divergence between the aggregated posterior and prior distribution. Despite the promising results, our work has some limitations. We have not conducted experiments on more complex architectures. This lack of exploration of advanced architectures may restrict the generalizability and scalability of our findings. In future work, we plan to extend our experiments to more sophisticated models and architectures to better understand the potential of DVAE in various scenarios.

## Figures and Tables

**Table 1:** Density estimation performance on Yahoo and Yelp datasets. The results are the mean values across 5 different random runs. ∗ indicates the results are referred from He et al. [12] and Shen et al. [20]. Hyper-parameters are listed in brackets and split by |if on different datasets. DVAE (wo) is the DVAE without KL weight dropout. DVAE (AB) is the DVAE without stopping strategy. VAE (w) is the VAE with KL weight dropout. Boldface denotes the best experimental results.

Model	Yahoo				Yelp			
	NLL	KL	MI	AU	NLL	KL	MI	AU
LSTM-LM	328.0	-	-	-	356.7	-	-	-
VAE	328.6	0.2	0.2	0.8	357.3	0.0	0.0	0.0
Cyclical	328.3	0.6	0.6	2.0	357.1	0.4	0.4	1.0
β-VAE_(0.4)_	328.5	6.5	6.1	6.8	357.4	6.1	5.9	4.0
FB_(0.1)_	328.3	3.4	2.4	32.0	357.0	4.8	2.6	32.0
BN-VAE_(0.6)_	326.9	6.5	5.8	32.0	**355.9**	6.7	5.8	32.0
DU-VAE_(0.6,0.8|0.5,0.8)_	326.9	8.8	7.2	28.0	356.0	7.4	6.6	15.0
δ-VAE_(0.1)_	329.7	3.2	0.0	2.0	357.9	3.2	0.0	0.0
MAE∗_(1.0,0.2|2.0,0.2)_	332.1	5.8	3.5	28.0	362.8	8.0	4.6	32.0
SA-VAE∗	327.2	5.2	3.7	9.8	**355.9**	2.8	1.7	8.4
Agg-VAE	326.7	3.6	3.6	12.0	356.0	3.0	2.9	10.0
EM-VAE	330.4	0.3	0.2	5.0	358.1	0.2	0.2	4.0
CNN-VAE∗	≤332.1	10.0	-	-	≤359.1	7.6	-	-
VAE-wh_(0.1)_	327.5	1.9	1.8	2.0	357.1	0.0	0.0	0.0
VAE-wh_(0.3)_	329.0	5.9	5.6	6.0	359.7	1.5	1.5	2.0
VAE (w)_(0.7)_	328.5	0.2	0.2	1.0	357.1	0.7	0.7	3.0
DVAE (wo)_(0.4|0.5)_	326.9	3.8	3.7	5.0	356.6	1.7	1.6	3.0
DVAE (AB)_(0.5,0.7)_	329.0	7.9	7.2	10.5	361.0	5.1	4.9	7.3
DVAE_(0.5,0.7)_	**326.5**	6.3	6.0	10.5	**355.9**	3.9	3.8	7.4

**Table 2:** Density estimation performance and classification accuracy with varying quantities of labeled data on the downsampled version of the Yelp sentiment dataset. ♯ Labeled-Data means the number of labeled data points. Hyper-parameters are listed in brackets. DVAE (wo) is the DVAE without KL weight dropout. Boldface denotes the best experimental results.

					Accuracy with ♯ Labeled-Data				
Model	NLL	KL	MI	AU	100	500	1 K	2 K	10 K
VAE	34.2	0.8	0.8	4.0	72.0	75.9	76.5	78.6	80.0
Cyclical	34.1	2.3	2.3	6.0	83.8	84.4	84.5	84.6	84.9
β-VAE_(0.7)_	34.1	2.7	2.6	2.0	59.3	59.8	59.6	59.5	60.0
BN-VAE_(0.6)_	**33.9 **	6.1	5.4	32.0	85.4	88.7	89.8	90.2	**90.4**
DU-VAE_(0.5,0.8)_	34.1	5.7	5.1	22.0	85.1	86.4	88.2	89.0	89.1
Agg-VAE	34.1	0.2	0.2	3.0	73.0	81.1	82.0	83.0	84.7
VAE-wh_(0.1)_	34.3	1.1	1.1	3.0	78.8	82.8	83.4	84.1	84.8
DVAE (wo)_(0.6)_	34.2	4.9	4.9	9.0	84.0	84.8	86.3	86.2	86.8
DVAE_(0.7,0.5)_	34.1	5.3	5.2	13.0	**89.5**	**89.8**	**90.2**	**90.3**	**90.4**

**Table 3:** Quality and diversity evaluation results of the generated texts on Yelp sentiment dataset. GPT2-P is the perplexity of the generated texts evaluated using GPT-2. DIST represents the mean value of the proportions of distinct 1-grams, 2-grams, and 3-grams in the generated texts. MAUVE measures the distribution closeness between the generated texts and human-written texts and is computed using GPT-2. Boldface indicates the best results within each group.

Model	SampleGen			InterpolateGen		
	GPT2-P	DIST_(e-3)_	MAUVE	GPT2-P	DIST_(e-3)_	MAUVE
VAE	75.81	0.77	0.01	74.81	0.69	0.01
Cyclical	119.46	**2.23 **	**0.10**	113.36	**2.06**	**0.08**
β-VAE_(0.7)_	132.44	1.70	0.05	121.80	1.57	0.04
Agg-VAE	**59.96**	0.29	0.01	**57.40**	0.27	0.01
VAE-wh_(0.1)_	85.59	1.09	0.02	86.71	0.99	0.01
BN-VAE_(0.6)_	156.45	**7.97**	**0.83**	150.19	7.34	0.58
DU-VAE_(0.5,0.8)_	151.26	7.48	0.80	145.96	7.12	0.54
DVAE(wo)_(0.6)_	**144.21**	6.99	0.82	**133.74**	6.71	0.57
DVAE_(0.7,0.5)_	148.92	7.90	**0.83**	137.84	**7.57**	**0.59**

## Data Availability

Publicly available datasets were used in this study. These data can be found here: https://github.com/SmilesDZgk/DU-VAE; https://drive.google.com/drive/folders/13sMpOJLFkROPxaIBKl8NUSOEvQjUQyq_ (accessed on 27 March 2025).

## Abbreviations

The following abbreviations are used in this manuscript:

DVAE	Dropout variational autoencoder
VAE	Variational autoencoder
KL	Kullback–Leibler divergence
LSTM	Long-short-term memory
